# Dimensions of Leisure and Perceived Health in Young University Students

**DOI:** 10.3390/ijerph17238750

**Published:** 2020-11-25

**Authors:** Montserrat Andrés-Villas, Diego Díaz-Milanés, Raquel Remesal-Cobreros, Mercedes Vélez-Toral, Pedro J. Pérez-Moreno

**Affiliations:** 1 Department of Social, Developmental and Educational Psychology, University of Huelva, 21004 Huelva, Spain; montserrat.andres@dpsi.uhu.es; 2 Department of Psychology, Universidad Loyola Andalucía, 41704 Sevilla, Spain; 3 Department of Clinical and Experimental Psychology, University of Huelva, 21004 Huelva, Spain; raquel.remesal@dpsi.uhu.es (R.R.-C.); pedro.perez@dpsi.uhu.es (P.J.P.-M.); 4 Mental Health Service at National System of Health, Juan Ramón Jiménez Hospital of Huelva, 21005 Huelva, Spain

**Keywords:** leisure, university students, health perception, healthy lifestyles

## Abstract

The aim of this study was to analyze the main leisure habits of students at the University of Huelva and the relationship with perceived health by grouping the various activities into components whilst also evaluating possible gender differences. The sample was selected through random cluster sampling and was composed of 903 students from various courses and degrees. Of the sample, 73.8% were female and 26.2% were male, with a mean age of 20.82 years. The participants responded to items measuring perceived health and the inventory of leisure activities extracted from the INJUVE (Spanish Youth Observatory) survey. The results obtained offered a solution of four components, grouping the activities into passive leisure, festive leisure, sports–competitive leisure and cultural leisure. Passive leisure was the most practiced and cultural leisure the least practiced. Statistically significant differences were observed between men and women in terms of the sports–competitive component and in the perception of health. In addition, a direct relationship was found between the sports–competitive dimension and health perception. These results support the existence of a “techno-active” profile in males and should be considered in the creation of university policies linked to health promotion or the prevention of risk behaviors.

## 1. Introduction

Leisure can be defined as “a form of using free time through a freely chosen and fulfilling occupation, whose very development is satisfactory or pleasant for the individual” [1]. In the 20th century, there was a growing interest in the study of leisure, with attempts to analyze the role it plays with respect to other dimensions of life. This evaluation established five dimensions of leisure, namely ludic, solidary, festive, creative and environmental–ecological, based on the purpose for which each one was practiced [2]. There are currently various instruments for evaluating leisure, such as the Questionnaire of Motivations, Attitudes and Behaviors in Youth Physical Sports Leisure (MACOFYD) developed by Ponce de León, Sanz, Ramos and Valdemoros [3], the INJUVE survey used since 2003 in the Survey of Opinion and Situation of Young People of the Youth Observatory in Spain [4], or questionnaires developed by various authors in order to conduct their studies. In most cases, the instruments do not evaluate the dimensions such as those referred to by Cuenca [2], limiting themselves simply to collecting information on preferences for and practice of different activities.

The study of leisure has yielded data such as those collected in the latest report of the Youth Observatory in Spain [5], which shows that the leisure activities most practiced by Spanish young people between 15 and 29 years old are firstly listening to music (93%), followed by going out with friends (92.7%), using the computer (88.8%), watching TV (77.9%), playing sports (78%) and resting/not doing anything (78%). The least practiced activities are attending conferences/colloquia (20.1%), going to the theater (26.7%) and going to museums or exhibitions (27%). With regard to gender differences, it can be observed that activities such as playing sports (83%/72.8%), using the computer (90.9%/86.5%) or playing video games (52.5%/17.4%) tend to be practiced by a greater proportion of males. Focusing on the university population and studying lifestyles, Rodriguez and Agulló observed that the activities most performed by university students were going out with friends (80.4%), going to discos/bars (70.3%), watching television (68.4%) or listening to the radio (68%) and the least performed activities were going to the movies (34.7%), cultural activities (33%) or playing sports (32%) [6].

In 1993, in the International Charter for Leisure Education [7], it was established that leisure promotes health and well-being and can be understood as a resource for personal, social and economic development; an important aspect of the quality of life; and a right. In addition, social and economic changes are giving rise to changes in leisure activities around the world, leading to the alleviation of dissatisfaction, stress, boredom and lack of creativity or physical activity, which are present in many current societies [7].

Leisure is considered a value [8,9] as well as being understood as one more dimension in relation to healthy lifestyles, with data indicating how beneficial it can be when it is put to good use [10]. In particular, Pascucci reports that engaging in leisure activities is related to how university students feel when they practice them, increasing the satisfaction they experience; making them feel happier and more positive; and contributing to the improvement of physical health, quality of life and well-being [11]. Likewise, it has been identified that leisure plays a relevant role in the promotion of development; the generation of relationships; the exchange of norms; the learning of behaviors and values; the acquisition of coping strategies; the generation of satisfaction; the development of skills related to cooperation, responsibility and communication; the generation of positive emotions; and an increase in the sense of mastery and competence as well as autonomy and self-control. One requirement for the generation of these positive effects is that leisure time is put to good use [11,12,13,14,15]. In addition, some data indicate that the practice of certain types of leisure activities such as reading or sports is positively associated with academic performance [16,17].

Regarding the relationship between leisure and health behaviors, Arrivillaga, Salazar and Correa found that the adequate management of leisure time is associated with a higher level of healthy behaviors [18]. They also found data to indicate that digital leisure or screen use is associated with the generation of high levels of satisfaction and entertainment [19,20], whilst sedentary habits are also associated with more irregular diets [21]. Given the scarcity of studies in this area, Kim and Brown point out that it would be interesting to study in greater detail the role played by types of leisure in determining health-related behaviors [22].

Further, most studies that relate leisure with health have focused on leisure as a sport/sedentary practice [23,24,25,26,27,28,29,30,31], such as the use of active video games [19,21,30] or alcohol/substance consumption [31,32], without providing information on the relationship that may exist with other types of leisure.

When considering gender differences, López, López, González and Fernández reported that activities such as playing sports, going out for a glass of wine, going out for drinks and playing board games are practiced more by males whilst a greater percentage of females watch television and remain sedentary [23]. In addition, some studies have identified in males what has been called a “techno-active profile” which consists of showing a high tendency to engage in physical activity and use video games [20,33,34]. Another line of research has emphasized the gap generated by the lack of time factor, identifying this as being a barrier in the practice of leisure that contributes towards maintaining gender differences, as in the case of physical activity, which is practiced more by males, compared with cultural activities, which are practiced more by females [35,36,37]. However, authors such as Bertuol et al. found no differences between Brazilian boys and girls aged 15–19 years [38].

Some ideas can be highlighted accordingly, such as that leisure is important in people’s lives, that there are changes in leisure practices, that leisure promotes health and well-being (despite the unclear relationship with healthy behaviors) and that there are differences in leisure practices between girls and boys. Based on these ideas and taking into account a sociocultural perspective, university students constitute a distinct group [6], and therefore it is of paramount importance to ascertain their leisure habits in order to devise the practices that entail the healthiest options. Thus, the objectives of this study were to analyze the possibility of grouping different activities into dimensions within the instrument of leisure evaluation, to identify the main leisure habits of students at the University of Huelva, to explore the possible relationship between leisure and perceived health and, finally, to identify any possible differences according to gender.

## 2. Materials and Methods

### 2.1. Participants 

Stratified random cluster sampling was conducted from the total number of degrees, courses and groups at the University. Firstly, the fields of knowledge of the offered degrees at the university were selected as strata (Art and Humanities, Engineering and Architecture, Natural Sciences, Health Sciences and Social and Legal Sciences) and the classrooms were selected as clusters. The number of participants selected between strata was proportional to the size of each one of them. Then, we randomly selected subjects of the first- and third-year degrees (e.g., Psychosocial Sciences from the first year of Bachelor’s Degree in Nursing) in each stratum.

The inclusion criteria for this study were that the students were enrolled in a degree of the University of Huelva and that they gave their explicit consent to participate in the study. The exclusion criteria were being an Erasmus student, being a student who had previously participated in the survey and being outside the age range of 18 to 29 years.

A total of 970 participants completed the questionnaire, of which 31 did not adequately complete the informed consent form, 35 did not fit into the age range and 1 did not indicate their gender; thus a total sample of 903 students was obtained, of which 73.8% were females and 26.2% were males, with a mean age of 20.82 years and a standard deviation of 2.422 years. The participants were distributed among the different subject areas as follows: Art and Humanities, 6.5%; Engineering and Architecture, 1.9%; Natural Sciences, 2.7%; Health Sciences, 39.6%; Social and Legal Sciences, 49.3%.

### 2.2. Variables and Instruments

For the sociodemographic data, an ad hoc questionnaire was used to collect information on the variables of age, gender and the university studies of the participants.

The leisure practices and their dimensions were studied by means of a scale extracted from the INJUVE [39], which is composed of 17 items with four response options ranging from “I do not carry out this activity” to “2 times a week or more” which evaluate the frequency with which various leisure activities are practiced. The original scale has 21 items, which, for this study, we reduced to 17 by regrouping the 3 that refer to reading (books, newspapers and magazines) and the 3 that refer to going to the movies, the theater and concerts. We have found no available scientific articles on the reliability of the instrument, following a literature search in the databases PsycARTICLES, PsycINFO and PsychologyDatabase, using “injuve AND (ocio OR leisure)”.

To measure perceived health, the indicator developed by Ilder and Benyamini [40] was used. This is an item with four response options: excellent, good, fair and poor.

### 2.3. Procedure

For the data collection, each of the selected classrooms was visited after an agreement with the corresponding lecturers. The data were collected in two different periods during the 2018/19 academic year. Both collection periods were performed under the same protocol for sampling, reaching the necessary sample size for a maximum error of estimation of 3% and a 95% confidence level according to the size of the population selected. The participating students were informed of the objectives of the study, as well as the voluntary nature of their participation and the anonymity of the data. After completing the questionnaires, the researchers, under the same protocol, proceeded to the digitalization phase. The research plan was based on cross-sectional explanatory relational analysis.

### 2.4. Data Analysis

We initially conducted a descriptive analysis of each of the variables and their differences according to gender. The principal component analysis (PCA) method was then used to extract the dimensions. Prior to this, the Kaiser–Meyer–Olkin (KMO) test for sampling adequacy and Bartlett’s test of sphericity were performed. The communities were studied, and the most parsimonious and adequate factorial solution was evaluated through parallel analysis (PA), on which a Promax rotation was applied. Following this, the Carmines and Zeller’s theta coefficient was obtained to evaluate the internal consistency and reliability of the scale.

Proportional contrast (χ^2^) and the respective analyses of adjusted standardized residuals and analysis of variance (ANOVA) were used to evaluate the possible relationships and differences between variables. The effect size of the differences and relationships found was calculated (contingency coefficient and partial eta squared).

For all cases, a statistical significance criterion of less than 5% (*p* < 0.05) was adopted. The statistical analyses were carried out using the statistical package IBM SPSS Statistics, version 23.0 (IBM, Armonk, NY, USA) [41].

### 2.5. Ethical Issues

This study has been approved by the Ethics Committee for Research Centers in Huelva (CEI) of the Andalusian Government with reference code 0846-N-19/P1027/19. In addition, the Declaration of Helsinki of 2013 [42] was taken into consideration, and the explicit permission of the participants was obtained through informed consent for the use and treatment of the data in a confidential and anonymous way. The data were kept by the research team.

## 3. Results

### 3.1. Descriptive Analysis of Leisure Practices, Health Perception and Differences According to Gender

Analysis of the leisure activities carried out by the university students revealed that the most practiced—at least once a week—were listening to music (93.53%), using the computer (92.53%), going out or meeting with friends (88.29%), watching television (82.67%) and resting or doing nothing (76.86%). The least practiced activities were attending conferences and talks (2.00%), going to museums and exhibitions (2.56%), traveling (4.46%), going on trips (6.11%) and attending sporting events (8.01%) (Table 1).

When evaluating leisure activities according to gender, statistically significant differences were found for playing sports (χ^2^(1; *N* = 847) = 71.516, *p* < 0.001; CC = 0.279), attending sporting events (χ^2^(1; *N* = 848) = 84.426, *p* < 0.001; CC = 0.301) and playing video games (χ^2^(1; *N* = 848) = 159.980, *p* < 0.001; CC = 0.398), all with a small effect size (Table 2), with males practicing all of these activities more than females (76.2% vs. 43.3%, 22.3% vs. 2.9% and 54% vs. 12.3%, respectively).

Moreover, 72.04%, 13.53%, 13.08% and 1.35% of the participants claimed to perceive their health as good, passable, excellent and poor, respectively, with statistically significant differences in the perception of health according to gender, with a negligible effect size (χ^2^(3; *N* = 887) = 27.476, *p* < 0.001; CC = 0.173).

**Figure 1:**
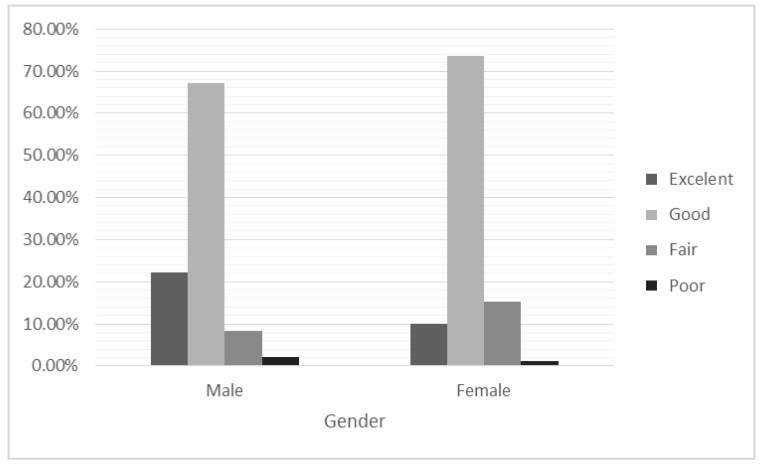
Percentages of perceived health ratings as a function of gender.

Adjusted standardized residuals showed that a significantly greater proportion of men perceived their health as excellent when compared with women (z = 4.7), whilst women perceived their health as good or fair to a greater extent than men, although the difference was smaller (z = 1.9 and z = 2.6, respectively). In this regard, a higher proportion of men responded as having poor health when compared with women (z = 1.3), with this difference being of a lower magnitude. These results can be seen in Figure 1, where more men rate themselves as having excellent/good health while women tend to indicate good/passable health.

### 3.2. Principal Component Analysis

First, the possibility of grouping the items used to evaluate leisure practices was analyzed. To this end, and to extract the dimensions of the data, a principal component analysis (PCA) was carried out. The results of the Kaiser–Meyer–Olkin test for sampling adequacy (KMO = 0.666) and Bartlett’s test of sphericity (χ^2^ (136) = 1841.867; *p* < 0.001) showed that the responses to the scale were adequate for further analysis.

Parallel analysis (PA) was then conducted to establish the number of components to be extracted, since this provides a statistical criterion for comparing eigenvalues. One thousand random simulations of the data matrix were used for this purpose, applying the PCA to each of them. The 95th percentile of the eigenvalues of each one of the components was then calculated, comparing the eigenvalue of each component of the observed matrix with its respective 95th percentile value of the random eigenvalues. Only one component was retained if its observed result was greater than this random result. The fifth observed component had a lower value than the 95th percentile of the fifth random component of the thousand matrices (fifth observed eigenvalue = 1.10 < 1.12 = fifth parallel eigenvalue). The first four observed components had a cumulative explained variance of 43.34% (Table 3).

An oblique rotation (Promax method) was then applied to these components. Table 4 displays the factorial loads of each item after eliminating those below 0.300.

The communalities indicated a good representation of the information in the extracted components except for the items referring to watching television (0.261); going to the cinema, theater or concerts (0.235); traveling (0.217); and resting or doing nothing (0.203).

The reliability of the reduction in dimensions, calculated by the Carmines and Zeller’s theta coefficient, suitable for dimensional reductions by PCA, was 0.626.

### 3.3. Descriptive Analysis of the Dimensions of Leisure and Differences According to Health Perception and Gender

Scores were generated for each component through the unweighted mean of the most saturated items in each one of them, once the rotation was applied. Component 1, named festive leisure, was composed of the items “Drinking, going out for drinks”, “Going to discos, dancing” and “Going out or meeting with friends”; Component 2, named sports–competitive leisure, was composed of “Playing sports”, “Attending sporting events” and “Playing with video games, consoles, etc.”; Component 3, named cultural leisure, was composed of “Going on trips”, “Traveling”, “Going to the cinema, theater, concerts, etc.”, “Going to museums, exhibitions”, “Attending conferences, talks” and “Reading books, newspapers, magazines”; and Component 4, named passive or rest leisure, comprised “Listening to music, CDs, tapes”, “Watching television”, “Listening to the radio”, “Using the computer” and “Resting, doing nothing”.

Analysis of leisure practices, based on the scores obtained according to each component, revealed that the most practiced type of leisure was classified as Component 4 (passive or rest leisure) with a mean score of 3.202 (SD = 0.445), followed by Component 1 (festive leisure) (M = 2.574; SD = 0.624), Component 2 (sports–competitive leisure) (M = 1.913; SD = 0.717) and, finally, Component 3 (cultural leisure) (M = 1.741; SD = 0.389).

Each of the dimensions of leisure was analyzed according to gender and health perception, with statistically significant differences found for sports–competitive leisure (Table 5).

The model explains 31% of the variance obtained in sports–competitive leisure (R^2^= 0.310; R^2corrected^ = 0.305). A statistically significant main effect of gender was observed for the component, with a moderate effect size (F (1,876) = 54.580; *p* < 0.001; η^2^_p_ = 0.059), with higher scores among men (Men = 2.366 > 1.634 = Women). Similarly, a statistically significant main effect of health perception was observed for the component, with a small effect size (F (3,876) = 9.962; *p* < 0.001; η^2^_p_ = 0.033).

**Figure 2:**
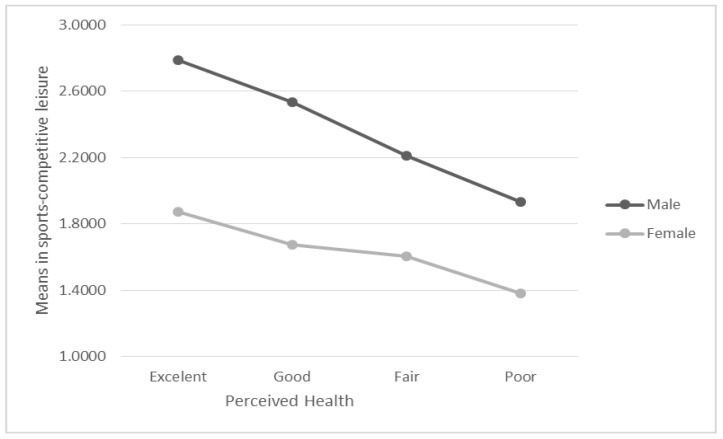
Mean perceived health scores according to gender for sports–competitive leisure.

These groups were compared with post hoc multiple tests using Bonferroni’s method, finding significant differences between the group that perceived their health to be excellent and the remaining groups (with *p* < 0.001, *p* < 0.001 and *p* = 0.001 when comparing this group with good, passable and poor, respectively) and between the good and passable health groups (*p* = 0.017). This indicates that this type of leisure is practiced less frequently in groups with poorer perceived health (see Figure 2).

However, no statistically significant interaction was found between perception of health and gender for sports–competitive leisure (F (3,876) = 1.271; *p* = 0.283).

## 4. Discussion

The main results of our study indicate that the leisure activities most practiced by the surveyed university students are listening to music, using the computer, going out or meeting with friends, watching TV, resting and playing sports. These findings are broadly similar to those presented in the latest report of the Institute of Youth with the exception of the practice of sport, which in this report was ranked in fifth place as opposed to sixth place in the case of university students [5]. In addition, it should be noted that the consumption of alcohol (drinking or going out for a drink) among university students is in eighth place, with 41.71% maintaining this practice at least once a week, and thus plays a more relevant role than in the case of the representative sample of young Spaniards of similar ages, for whom this activity is ranked in thirteenth place, with 20% maintaining this practice at least once a week [5]. These data are worrying and should be taken into account, particularly for health promotion actions that can be programmed from the university, and could also help to inform the design of studies that allow for identifying the factors linked with the maintenance and increase of this risk behavior in this population. In contrast, we found that activities such as traveling or listening to the radio are less frequently practiced by university students.

With regard to other less practiced activities, we found similarities with those included in the report of the Youth Institute [5] in attending conferences or colloquia, visiting museums or exhibitions, going on trips and attending sporting events. Further, if we compare these data with those published approximately a decade ago by Rodríguez and Agulló, we can observe how leisure practices have changed, with activities such as playing sports, listening to music or using the computer being more predominant and overtaking others such as going to a discotheque, the cinema or listening to the radio, which are now among the least practiced activities [6]. We can therefore see how young university students have increased the practice of sports as part of their leisure. These data seem relevant to us, considering the importance attributed to physical activity in relation to future health [43,44]. However, the gender differences found here to indicate the lower practice of physical activity in females is a worrying result that is in line with the findings reported in other studies on leisure [23,45], in the study on physical activity in adolescents commissioned by the WHO [46] or in the study on physical activity in Spanish university students by Práxedes et al. [47].

Further, in relation to the evaluation of leisure, we have conducted principal component analysis of the responses to the questionnaire used, to assess the existence of dimensions that allow us to establish the type of leisure beyond the mere identification of the more or less practiced activities. Our data have revealed the presence of four components that we have termed festive leisure (Component 1), sports–competitive leisure (Component 2), cultural leisure (Component 3) and passive or rest leisure (Component 4). The reliability obtained was 0.626 and the variance explained by the four main components was 43.342%. Cuenca’s concept of leisure included the ludic, festive, creative, solidary and environmental–ecological dimensions; in our case, we observed that the obtained components coincide with the festive dimension, the cultural component forms part of what Cuenca termed creative leisure and the ludic dimension corresponds with the sports–competitive component, whilst the solidary and environmental–ecological dimensions were not expected to appear because the questionnaire did not contain any items related to these aspects [2]. In addition, the sports–competitive dimension reflects the trend identified by other authors who point to the presence of physical activity/sedentarism linked to the use of screens or the Internet as modes of leisure [48,49], which has been termed the “techno-active” profile [33,50,51]. The data related to this dimension are also in line with the appearance and increase in leisure activities linked to the use of new technology or screens in general that has been witnessed in recent years. It has also been noted that this type of leisure activity is practiced more by males than females [49,52], which also coincides with our results. In short, our analysis of the main components allows us to state that through this questionnaire we can obtain information about four dimensions in which leisure time is used, identify which of these are more predominant and contemplate relevant aspects of the current leisure trends among young people.

In relation to the dimensions of leisure that are located at the extremes, at one end we can find passive leisure to be the type that is most frequently practiced, whilst at the opposite end is cultural leisure. These data are in line with the previously mentioned studies that point to the maintenance of a more sedentary lifestyle [46,47], as well as a lower frequency of involvement in activities linked to cultural exhibitions among the young population [5,6]. As hypotheses for future studies, it could be suggested that economic factors could explain the infrequent practice of cultural activities and the more predominant role of passive activities, which could be more accessible. 

The results of our study also indicate that the perception of health is related to the sports–competitive dimension of leisure in that the greater practice of this type of leisure is associated with a better perception of health. A relationship was also found with gender, observing how most of the females reported their health as being good/passable whilst the males reported their as excellent/good. These variables explain 31% of the practice of this type of leisure. To date, studies have revealed the relationship between the perception of health and leisure activities linked to physical activity or a sedentary lifestyle [23,24,25,26,27,28,29,30,31,43] and also the role of the use of active video games in health [19,21,30]. However, there appears to be no available data from other studies that indicate the relationship between this techno-active or sports–competitive profile and health, or the effect of gender on this relationship. Thus, our results may be of relevance despite the fact that there is a need for a more in-depth investigation into the factors that give rise to this balance in favor of greater perceived health, such as the fact that a greater use of technology could be associated, as Zach suggests, with a higher level of physical activity, which has a positive effect on health [48].

## 5. Conclusions

In conclusion, it can first be stated that the questionnaire used allows us to obtain reliable information on leisure practices linked to the dimensions of passive leisure, festive leisure, sports–competitive leisure and cultural leisure.

The most practiced leisure activities were listening to music, using the computer, going out to meet friends or watching TV. The least practiced were attending conferences, traveling, going on trips, attending sports events or going to the disco to dance.

In addition, the data obtained allow us to conclude that there is a general increase in leisure linked to alcohol consumption regarding the general population of similar characteristics and that males spend more of their free time engaged in physical leisure activities, attending sporting events and playing video games when compared with females. There are also differences in health perception between boys and girls, with boys having better self-perceived health.

Finally, regarding the dimensions of leisure, passive leisure is the type that is most frequently practiced, and statistically significant differences were observed between males and females in the sports–competitive dimension. Finally, it should be noted that the perception of health is related to the sports–competitive dimension.

The data found may be useful for informing the development of university policies linked to health promotion or the prevention of risk behavior. In this regard, it is worth noting the differences that should be considered in relation to gender, along with the positive link found between the perception of health and the sports–competitive dimension.

## 6. Strengths and Limitations

Strengths of this study include the sample size and the representativeness of students from the University of Huelva, due to the fact that the participants were recruited by stratified cluster sampling.

In addition, the data obtained allow us to continue advancing in the knowledge of the role played by leisure practices play in well-being and their relationship with other health-related behaviors, whilst identifying different aspects linked to gender along with issues that require further investigation.

Further, grouping the practices into dimensions has allowed us to obtain information based on categories that group combined activities to a greater or lesser extent, which could help to establish profiles related to leisure practices. In addition, this facilitates the identification of groups of practices that could be linked to health and that could also differ according to gender. These data could help to guide us towards future lines of action for further, more in-depth investigations.

Among the main limitations of our study, we must first note the cross-sectional nature of our study design, which does not allow us to obtain information on the evolution of the behaviors studied throughout the course of university life.

To the best of our knowledge, this is the first study aimed at assessing the properties of the leisure inventory developed by the INJUVE and its suitability to be used as a multidimensional measurement tool. However, further research about these properties in different contexts is needed to generalize the obtained results and fill the current gap.

With regard to leisure activities related to alcohol consumption, as well as the practice of physical activity, our data do not provide information on the frequency and quantity of alcohol consumption or on the intensity and frequency of physical activity, which are aspects worthy of consideration in future studies.

Further, in relation to the perception of health, the low number of participants who have reported their perception of health as being very poor does not allow us to observe any significant differences in that category, and thus we have less information in this regard.

Finally, the characteristics of the sports–competitive dimension require further study, particularly of the role played by the various components linked associated with this dimension, as well as consideration of other variables that have not been considered in this study, such as the characteristics of the physical activity practiced, the time spent per day or per week on the various activities or the reasons (including those of an economic nature) for practicing (or not) the activities indicated.

## Figures and Tables

**Table 1:** Activity performance percentages.

Activity	Does Not Carry Out or Performs 2–3 Times per Month	Performs 1 or More Times per Week
Drinking, going out for drinks	58.29%	41.71%
Going to discos, dancing	72.86%	27.14%
Going out to meet friends	11.71%	88.29%
Doing sport	48.00%	52.00%
Attending sporting events	91.99%	8.01%
Going on trips	93.89%	6.11%
Traveling	95.54%	4.46%
Going to the cinema, theater, concerts, etc.	86.16%	13.84%
Listening to music, CDs, tapes	6.47%	93.53%
Going to museums and exhibitions	97.44%	2.56%
Attending conferences and talks	98.00%	2.00%
Reading books, journals, magazines	49.44%	50.56%
Watching television	17.33%	82.67%
Listening to the radio	68.12%	31.88%
Using the computer	7.47%	92.53%
Playing video games, consoles, etc.	77.09%	22.91%
Resting, doing nothing	23.14%	76.86%

**Table 2:** Differences in leisure activities according to gender.

Activities	χ^2^	*p*	CC
Drinking, going out for drinks	0.307	0.579	
Going to discos, dancing	0.512	0.474	
Going out to meet friends	0.006	0.938	
Playing sports	71.516	<0.001	0.279
Attending sporting events	84.426	<0.001	0.301
Going on trips	0.863	0.353	
Traveling	1.538	0.215	
Going to the cinema, theater, concerts	0.220	0.639	
Listening to music, CDs, tapes	0.516	0,473	
Attending museums, exhibitions	0.001	0.974	
Attending conferences and colloquia	0.682	0.409	
Reading books, journals, magazines	1.403	0.236	
Watching television	1.279	0.258	
Listening to the radio	0.388	0.533	
Using the computer	0.362	0.547	
Playing video games, consoles, etc.	159.98	<0.001	0.398
Resting, doing nothing	0.130	0.719	

**Table 3:** Eigenvalues of extracted components.

Initial Eigenvalues			
Components	Total	% Variance	% Cumulative
1	2.433	14.310	14.310
2	2.123	12.490	26.800
3	1.450	8.529	35.329
4	1.362	8.013	43.342
5	1.103	6.488	
6	1.049	6.173	
7	0.920	5.411	
8	0.898	5.283	
9	0.830	4.883	
10	0.794	4.672	
11	0.723	4.254	
12	0.662	3.894	
13	0.648	3.810	
14	0.594	3.492	
15	0.573	3.372	
16	0.487	2.863	
17	0.351	2.063	

**Table 4:** Configuration matrix following Promax rotation.

Variable	1	2	3	4
Drinking, going out for drinks	0.855			
Going to discos, dancing	0.840			
Going out to meet friends	0.700			
Playing sports		0.606		
Attending sporting events		0.785		
Playing with videogames, consoles, etc.		0.687		
Going on trips			0.602	
Traveling			0.628	
Going to the cinema, theater, concerts, etc.			0.425	
Going to museums, exhibitions			0.750	
Attending conferences, colloquia			0.650	
Reading books, journals, magazines			0.499	
Listening to music, CDs, tapes				0.449
Watching television				0.640
Listening to the radio				0.335
Using the computer				0.632
Resting, doing nothing				0.434

**Table 5:** ANOVA models for the components of leisure.

Dimension	F	*p*	η^2^_p_
Festive leisure	1.115	0.351	0.009
Sports–competitive leisure	56.348	<0.001	0.310
Cultural leisure	1.290	0.252	0.010
Passive or rest leisure	0.750	0.629	0.006
